# Differential expression of lymphocyte subpopulations in the peripheral blood of patients with COVID‐19: Implications for disease severity and prognosis

**DOI:** 10.1002/iid3.1281

**Published:** 2024-05-23

**Authors:** XinQiang Xu, JunYuan Huang, Haiqi Zhang, Weiguo Lu, Jiduo Liu

**Affiliations:** ^1^ The First Affiliated Hospital University of Chinese Medicine Guangzhou China; ^2^ Guangdong Clinical Research Academy of Chinese Medicine Guangzhou China; ^3^ Guangzhou United Yijian Medical Laboratory Co., Ltd Guangzhou China

**Keywords:** animals, B cells, cells T Cells, cells viral/retroviral, human, infections

## Abstract

**Objective:**

To investigate the expression patterns and clinical significance of specific lymphocyte subsets in the peripheral blood of patients with severe acute respiratory syndrome coronavirus 2 (SARS‐CoV‐2) infection.

**Methods:**

Between December 2022 and February 2023, a cohort of 165 patients from the First Affiliated Hospital of Guangzhou University of Chinese Medicine were analyzed. The participants represented various stages of coronavirus infection severity: mild, moderate, severe, and critical. Additionally, 40 healthy individuals constituted the control group. The FC 500MPL flow cytometer and associated reagents for flow cytometry.

**Results:**

Compared with the healthy control group, activated B lymphocytes witnessed a pronounced increase (*p* < .05). A significant decrease was observed in the levels of Breg, Cytotoxic T cells or Suppressor T‐cell (Tc/s), late‐activated T, late‐activated Th, and late‐activated Tc/s lymphocytes (*p* < .05). Th, initial Th, initial Tc/s, total Treg, natural Treg, induced Treg, early activated T, and early activated Th lymphocyte levels showed no significant difference (*p* > .05). As the disease progressed, there was an uptick in midterm activated T lymphocytes (*p* < .05), while Breg, T, Tc/s, senescent Tc/s, and total senescent T levels dwindled (*p* < .05). Noteworthy patterns emerged across different groups for B1, T‐lymphocytes, Tc/s, B2, CD8+ Treg cells, and other subsets, highlighting variance in immune responses relative to disease severity. When juxtaposed, no significant difference was found in the expression levels of lymphocyte subsets between patients who died and those deemed critically ill (*p* > .05).

**Conclusion:**

Subsets of Treg and B‐cells could act as yardsticks for the trajectory of SARS‐CoV‐2 infection and might have potential in forecasting patient trajectories. A comprehensive evaluation of lymphocyte subsets, especially in real‐time, holds the key to discerning the clinical severity in those with COVID‐19. This becomes instrumental in monitoring treatment outcomes, tracking disease evolution, and formulating prognostications. Moreover, the results provide a deeper understanding of the cellular immune defense mechanisms against the novel coronavirus infection.

## Graphical abstract

We investigated the expression patterns and clinical significance of specific lymphocyte subsets in the peripheral blood of patients with severe acute respiratory syndrome coronavirus 2 infection.

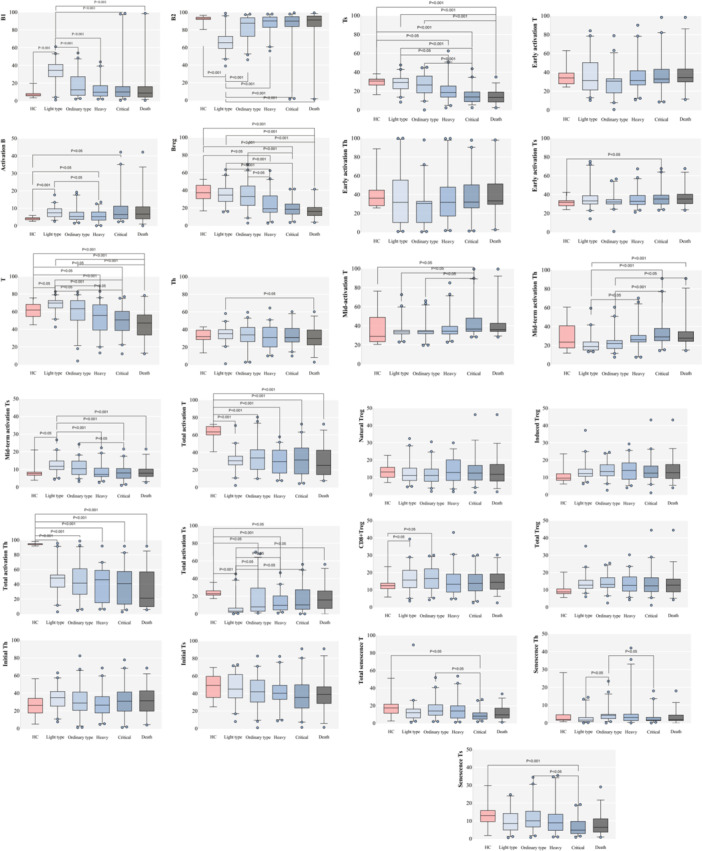

## 1 INTRODUCTION

The Omicron variant in the novel coronavirus has gradually become the dominant global epidemic strain since 2022, and its transmissibility and immune evasion ability have been significantly enhanced, but its pathogenicity has been significantly weakened.^1^ After infection with the new coronavirus, there are different clinical manifestations and types, which are closely related to immune function. Recent studies have highlighted a concerning trend in patients with coronavirus disease 2019 (COVID‐19): a marked reduction in the count of peripheral blood lymphocytes, especially for severe cases.^2^ This encompasses subsets such as CD4 + T, CD8 + T, and B cells.^3^, ^4^, ^5^ Of particular concern is the sharp decrease in CD8 + T cells seen in patients grappling with severe COVID‐19.^6^ Intriguingly, a decline in CD8 + T cells a week after starting treatment suggests diminished therapeutic effectiveness.^7^ Moreover, CD8 + T‐cell counts that dip to ≤ 75 cells/μL have been independently linked with higher mortality rates and are now considered potent indicators of patient prognosis.^8^ A higher count of virus‐specific CD8 + T cells often point to a more optimistic patient outcome.^9^, ^10^ In stark contrast to their mildly affected counterparts, severely symptomatic patients‐especially those in intensive care‐exhibit a significantly reduced total of peripheral blood lymphocytes. This includes reductions in T, CD4 + T, and especially CD8 + T cells. Clinical data has pinpointed a relationship between the quantity of COVID‐19‐specific T cells in lung tissue and enhanced clinical protection.^11^ Moreover, these specific memory T cells have been detected as long as 10 months after the initial infection,^12^ emphasizing the crucial role lymphocytes play in dictating the severity of a COVID‐19 case. Interestingly, a majority of patients with COVID‐19 (although not universally) also present with an increased count of either HLA‐DR + CD38 + ‐activated T cells or CD71 + ‐activated T cells. In summary, the prevailing observation is that patients with COVID‐19 typically have decreased peripheral blood lymphocyte counts, accompanied by a pronounced activation of the T‐cell phenotype.

In summary, this study can help determine the classification and diagnosis of the severity of clinical condition of patients with COVID‐19 through real‐time detection of fine subsets of lymphocytes, which has certain clinical significance for the evaluation of treatment effect, disease progression, and prognosis, and can also provide scientific basis for the pathogenesis of new coronavirus infection in cellular immunity.

## 2 MATERIALS AND METHODS

### 2.1 Sample collection and inclusion criteria

Study Groups: Participants were categorized into six groups: healthy control (40 participants), mild COVID‐19 (41 participants), common COVID‐19 (43 participants), severe COVID‐19 (two sub‐groups: 41 participants and 40 participants), and the death patients from each group of COVID‐19 infection were summarized to form the death group (a total of 34 cases). Severity of COVID‐19 infection was graded according to China National Health Commission Guidelines for Diagnosis and Treatment of COVID‐19 infection (tenth version). Laboratory‐confirmed patients with upper respiratory tract infection as the main manifestation were considered mild cases. Laboratory‐confirmed patients with an oxygen saturation of >93% and a new crown pneumonia on imaging were considered moderate cases. Laboratory‐confirmed patients who met an oxygen saturation of ≤93% and had imaging to show new coronary pneumonia were considered to have severe COVID‐19: (1) respiratory distress (respiration rate, ≥30/min), (2) resting oxygen saturation ≤93%, or (3) arterial oxygen partial pressure (PaO_2_)/fraction of inspired oxygen (FiO_2_) ≤ 300 mmHg (1 mmHg = 0.133 kPa). Patients who had any of the following were considered critically ill: (1) respiratory failure requiring mechanical ventilation, (2) shock, or (3) failure of other organs requiring intensive care unit admission.

Inclusion criteria:

(1) Diagnosis based on the established criteria for COVID‐19.

(2) Both participants and their families must comprehend the study objectives and provide signed informed consent.

(3) Must have obtained approval from the hospital's ethics committee.

Exclusion criteria:

(4) Concurrent infection with other viruses or mycoplasma.
(5) Presence of other acute infectious diseases.
(6) Any participant removal due to disagreements or external complications.

Control: A total of 23 healthy controls were included in this study. Those with any disease symptom (such as fever, cough, obstruction, or other unwellness) that indicated the infection or those with any immunocompromised disease were excluded from the control group.

Biomarkers Analyzed: The study analyzed markers such as B_1_, B_2_, activated B, Breg, Treg, including their subsets, various activated T cells, and aging T lymphocytes.

### 2.2 Equipment and reagent selection

Reagents used: The following cell solutions were used for detection: B1, B2, activated B lymphocytes (CD10, PE/CD5, PC7/CD19, and ECD), Breg lymphocytes, Treg subpopulation, aging activated T lymphocytes, and advanced activated T lymphocytes. Additionally, phosphate‐buffered saline (PBS) and FLOW CHECK flow microsphere solutions were incorporated.

Instrumentation: We employed the FC‐500 MPL flow cytometer manufactured by Beckman Coulter.

### 2.3 Experimental procedure

Detection: The FC‐500 MPL flow cytometer was the primary instrument. FLOW CHECK microspheres were used for quality assurance. Each test utilized a designated flow cytometry tube. The process encompassed the sequential addition of specific antibodies, anticoagulated whole blood, red blood cell lysate, and PBS solution.

Gating Methods: For specific gating methods applied to different cells. B1 (CD5 + CD19 + ), B2 (CD5‐CD19 + ), activated B (CD10 + CD19 + ), regulatory B lymphocytes (CD19 + CD24 + CD38 + ); The T lymphocyte subpopulations include initial T, regulatory T, activated T, and aging T cells. The initial T cells include initial Th cells (CD3 + CD4 + CD45RA + ), initial Tc/s cells (CD3 + CD8 + CD45RA + ), total Treg (CD3 + CD4 + CD25+CD127low), natural Treg (CD3 + CD4 + CD25+CD127low CD45RA + ), inducible Treg (CD3 + CD4 + CD25+CD127low CD45RA‐), and CD8+Treg (CD3 + CD8 + CD25+CD127low). The activated T cells include early activated T cells (CD3 + CD69 + ), early activated Th cells (CD3 + CD4 + CD69 + ), early activated Tc/s cells (CD3 + CD8 + CD69 + ), Midterm activated T cells (CD3 + CD25 + ), Midterm activated Th cells (CD3 + CD4 + CD25 + ), Midterm activated Tc/s cells (CD3 + CD8 + CD25 + ), late‐activated T cells (CD3 + HLA‐DR + ), late‐activated Th/s cells (CD3 + CD4 + HLA‐DR + ), and late‐activated Tc/s cells (CD3 + CD8 + HLA‐DR + ). Senescence T cells include total senescence T cells (CD3 + CD57 + ) and senescence Th cells. (CD3 + CD4 + CD57 + ), senescence Tc/s Cells (CD3 + CD8 + CD57 + ).

### 2.4 Data analysis

Software used: Data analysis was conducted using SPSS 20.0 and GraphPad Prism 9.0.

Statistical tests: The Shapiro–Wilk test determined data normality, followed by the independent‐samples *t*‐test and the nonparametric Kruskal–Wallis rank sum test. Results with a *p*‐value below .05 were considered significant.

## 3 RESULT

### 3.1 Patient demographics

The study encompassed 165 adult patients. We observed distinct differences in age and gender among groups. Older males predominantly characterized the severe and critically ill patient groups, in contrast to the milder cases. Detailed age breakdown of the severe acute respiratory syndrome coronavirus 2 (SARS‐CoV‐2) infection group and the control group is shown in Table 1.

**Table 1:** Basic information and clinical typing in the two groups (cases).

Group	Age (years)	Gender	
		Male	Female
Light and ordinary (A)	57 (37, 76)	44	40
Heavy and critical (B)	80 (71, 86)	62	19
Control	39 (22, 53)	9	14
*p*‐value (A vs. B)	.000	.001	

### 3.2 Comparison of lymphocyte subsets

#### 3.2.1 Findings

Patients with COVID‐19 displayed increased activated B lymphocyte levels compared with healthy controls, while several other lymphocyte levels were diminished.

Disease severity correlated with fluctuating levels of activated T, Breg, T, Tc/s, aging Tc/s, and total aging T cells.

A meticulous analysis between the groups showcased unique patterns for different lymphocyte subsets, as elaborated further in our study. For detailed results, please refer to Table 2 and Figure 1.

**Table 2:** Comparison of lymphocyte subsets between the healthy control and COVID‐19‐infected groups(%) [M(p25, p75)/x ± s].

Projects	Healthy control group (*N* = 40)	Light‐type group (*N* = 41)	Ordinary‐type group (*N* = 43)	Heavy group (*N* = 41)	Critical group (*N* = 40)	Death group (*N* = 34)
B_1_	6.70 (5.40, 8.40)	32.67 ± 12.79^a^	12.40 (5.90, 27.53)^b^	9.80 (5.20, 17.30)^b^	17.42 ± 23.55^b^	8.75 (3.98, 16.25)^b^
B_2_	93.30 (91.60, 94.60)	67.33 ± 12.79^a^	87.60 (72.48, 94.10)^b^	90.20 (82.70, 94.80)^b^	82.58 ± 23.55^b^	91.25 (83.75, 96.03)^b^
Activation B	4.08 ± 0.90	7.68 ± 3.37^a^	5.30 (3.48, 7.95)	5.69 ± 3.39^a^, ^b^	6.45 (3.85, 11.35)^a^	6.65 (3.70, 10.75)
Breg	36.39 ± 10.94	35.22 ± 10.62	33.24 ± 15.07	24.09 ± 13.74^a^, ^b^, ^c^	18.40 (12.75, 24.63)^a^, ^b^, ^c^	16.25 (11.20, 21.03)^a^, ^b^, ^c^
T	61.11 ± 8.67	67.83 ± 8.11^a^	60.00 ± 16.24^b^	52.76 ± 17.23^a^, ^b^	49.87 ± 15.14^a^, ^b^, ^c^	44.94 ± 16.97^a^, ^b^, ^c^
Th	32.43 ± 7.76	35.24 ± 9.70	32.91 ± 12.45	31.13 ± 13.23	33.55 ± 11.26	30.04 ± 12.81^b^
Ts	29.3 ± 5.42	29.21 ± 8.08	26.65 ± 10.68	18.60 (13.45, 25.65)^a^, ^b^	14.94 ± 8.25^a^, ^b^, ^c^	13.89 ± 7.27^a^, ^b^, ^c^
Early activation T	34.00 (27.60, 39.40)	37.11 ± 19.05	29.91 ± 14.86	31.40 (26.45, 41.85)	32.80 (28.63, 43.38)	34.30 (29.63, 43.63)
Early activation Th	36.10 (27.90, 44.80)	36.94 ± 29.27	29.12 ± 20.96	31.70 (16.90, 48.05)	31.90 (25.63, 50.43)	33.15 (29.33, 51.48)
Early activation Ts	31.26 ± 4.91	35.89 ± 12.02	33.26 ± 9.18	34.58 ± 8.78	37.3 ± 10.88^a^	35.20 (30.05, 40.53)
Mid‐activation T	29.00 (23.20, 49.00)	35.07 ± 9.35	33.90 (31.60, 35.32)	34.40 (31.30, 39.65)	43.05 ± 16.2^a^, ^b^, ^c^	36.05 (33.95, 42.80)
Midterm activation Th	23.50 (17.10, 41.00)	21.64 ± 9.26	23.22 ± 10.33	28.10 ± 12.20^b^	34.38 ± 16.49^b^, ^c^	27.70 (24.08, 34.70)^b^, ^c^
Medium‐term activation Ts	7.70 (6.60, 8.60)	12.63 ± 4.37^a^	10.92 ± 4.61	7.20 (5.85, 10.80)^b^	8.20 ± 4.24^b^, ^c^	7.90 (5.95, 10.30)^b^
Total activated T	63.50 (59.80, 69.90)	30.09 ± 11.85^a^	33.70 (20.08, 43.38)^a^	29.50 (16.15, 42.80)^a^	31.73 ± 18.78^a^	25.15 (14.40, 42.70)^a^
Total activation Th	94.37 ± 1.52	48.40 (35.90, 52.75)^a^	41.50 (26.05, 59.78)^a^	46.20 (14.80, 59.15)^a^	36.46 ± 26.16^a^	21.10 (9.45, 57.03)^a^
Total activation Ts	23.92 ± 4.45	3.10 (1.65,6.80)^a^	16.74 ± 18.47^a^, ^b^	13.16 ± 10.58^a^, ^b^	16.41 ± 14.82^a^, ^b^	15.77 (5.95, 26.18)^b^
Initial Th	27.05 ± 14.43	34.39 ± 13.65	31.06 ± 16.3	28.44 ± 16.09	30.96 ± 17.99	32.03 ± 15.99
Initial Ts	47.17 ± 13.89	46.20 ± 16.44	41.93 ± 17.19	41.68 ± 16.29	38.55 ± 21.86	40.42 ± 20.24
Natural Treg	13.37 ± 4.43	12.66 ± 6.29	12.08 ± 6.03	14.18 ± 7.36	14.13 ± 8.84	11.80 (7.75, 17.58)
Induced Treg	9.60 (8.20, 12.20)	13.63 ± 5.60	13.95 ± 5.03	14.16 ± 6.07	13.84 ± 6.81	12.65 (9.25, 17.53)
CD8+Treg	12.43 ± 3.91	16.35 ± 6.97^a^	16.63 ± 7.10^a^	14.51 ± 8.11	14.34 ± 6.80	14.85 ± 6.29
Total Treg	8.80 (7.70, 10.60)	13.61 ± 5.07	13.88 ± 5.12	13.73 ± 5.66	13.74 ± 7.30	12.70 (8.73, 16.28)
Total senescence T	17.30 (11.10, 21.70)	11.80 (5.80, 16.25)	13.80 (8.50, 20.65)	13.80 (5.60, 19.95)	8.00 (4.35, 11.98)^a^, ^c^	9.40 (5.60, 17.25)
Senescence Th	2.00 (1.40, 4.70)	1.60 (0.65, 3.20)	4.30 (2.28, 4.88)^b^	3.10 (1.20, 5.00)	1.60 (1.20, 3.25)^c^	1.95 (1.20, 4.52)
Senescence Tc/s	13.27 ± 7.25	8.60 (4.75, 14.25)	10.00 (6.38, 16.18)	8.90 (4.50, 13.80)	6.80 ± 5.06^a^, ^c^	6.45 (3.58, 11.28)

Abbreviations: COVID‐19, coronavirus disease 2019; SARS‐CoV‐2, severe acute respiratory syndrome coronavirus 2.

^a^ Healthy control group compared with each group of SARS‐CoV‐2 infection, *p*＜.05.

^b^ Light‐type group compared with normal‐type group, heavy‐type group, critical‐type group, and death group, *p*＜.05.

^c^ Ordinary‐type group compared with heavy‐type group, critical‐type group, and death group, *p*＜.05.

^d^ Heavy‐type group compared with critical‐type group and death group, *p*＜.05.

^e^ Critical‐type group compared with death group, *p*＜.05.

Figure 1Boxplots comparing the healthy control group with each group of severe acute respiratory syndrome coronavirus 2 (SARS‐CoV‐2) infection and each index between SARS‐CoV‐2 infection groups (%).

**Figure:**
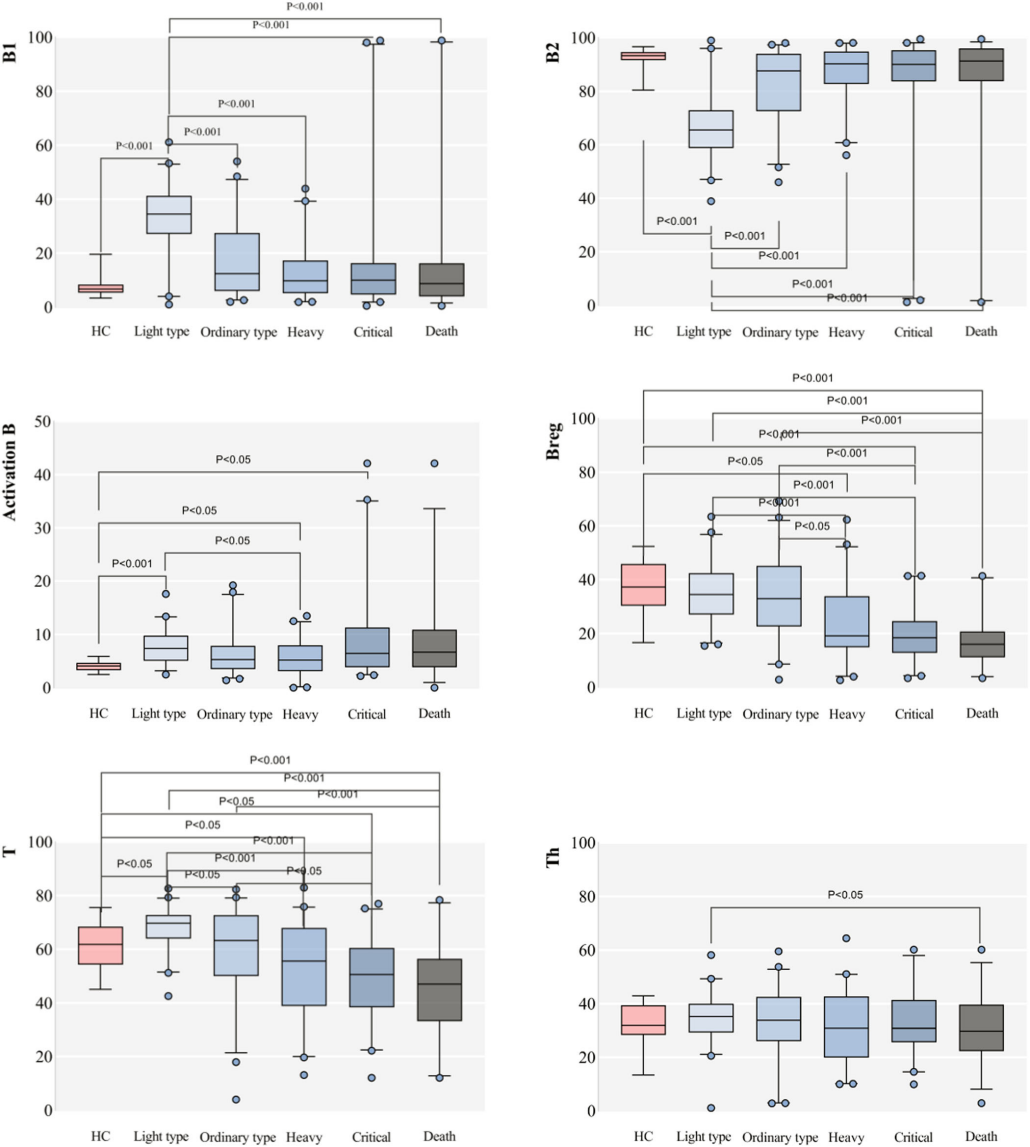

**Figure:**
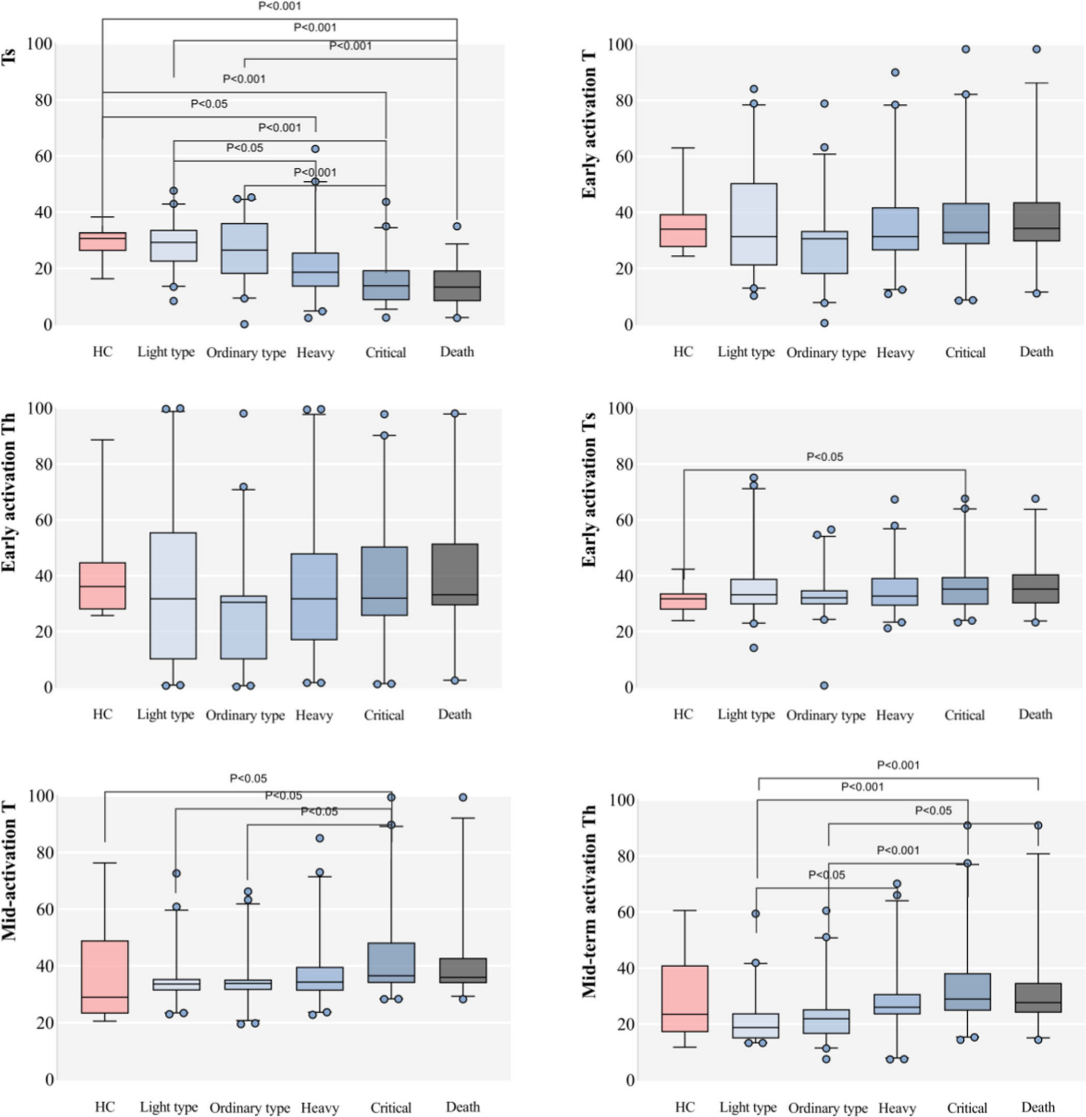

**Figure:**
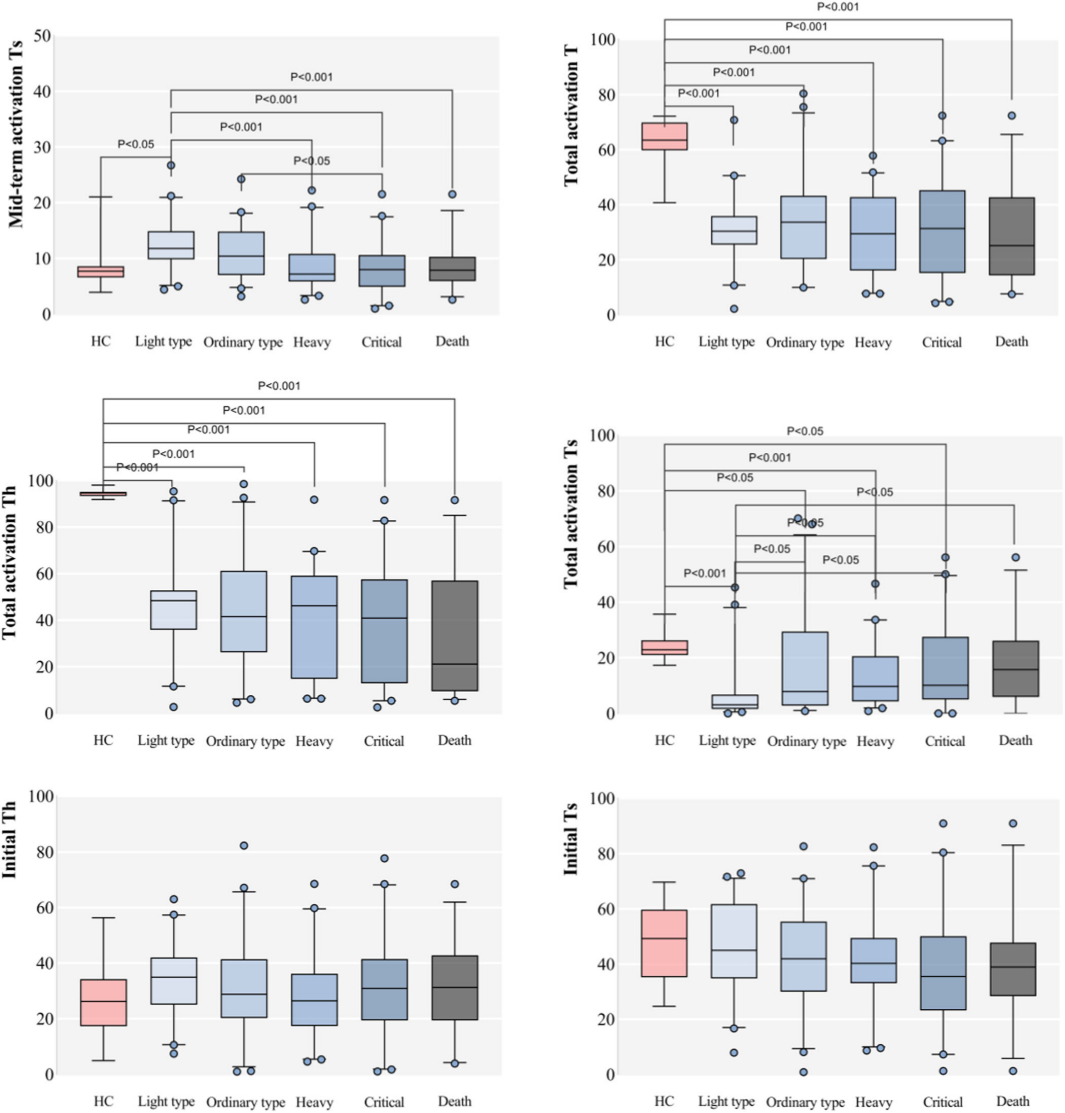

**Figure:**
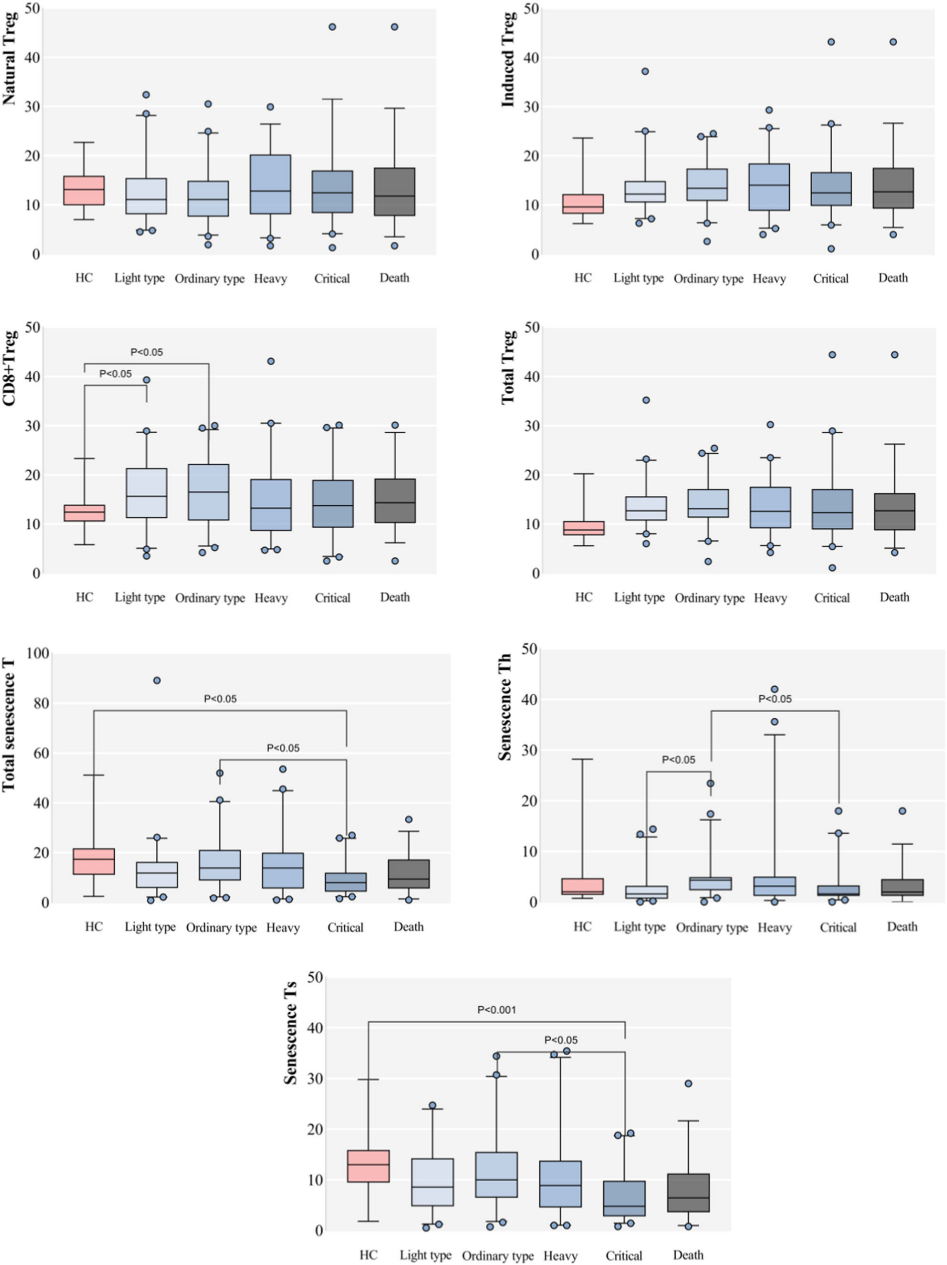

## 4 DISCUSSION

While most patients with COVID‐19 experience mild to moderate symptoms, a subset may progress rapidly to severe or critically severe stages, elevating the risk of mortality. A substantial reduction in peripheral blood lymphocyte counts in these patients, signifying considerable lymphocyte damage caused by COVID‐19. This, in turn, compromises the body's immune function, potentially exacerbating the patient's condition and leading to fatal outcomes. Thus, lymphocyte depletion may serve as a crucial factor in the deterioration of the patient's status. Thus, the extent of this depletion could serve as an important indicator for assessing mortality risks.

In our study, we found notable disparities in age and sex among different COVID‐19 patient groups (*p* < .05). The severe and critical groups consisted of older and predominantly male patients compared with the mild and ordinary groups. This finding highlights that older patients with COVID‐19 may be more vulnerable to severe forms of the disease, emphasizing age as a critical risk factor. Alongside lung complications, as the disease advances, patients experience inflammatory reactions in other organs, such as the heart, liver, and kidneys. The severity of these organ damages aligns with the disease's clinical progression. While patients with mild disease might present heightened levels of specific inflammatory markers, those with severe symptoms often showcase increased levels across most inflammation indicators, resulting in more pronounced organ injuries. In comparison to healthy individuals, patients with COVID‐19 had a significant surge in activated B lymphocyte levels (*p* < .05). By contrast, levels of Breg, Tc/s, and various late‐activated T, late‐activated Th, and late‐activated Tc/s lymphocytes were considerably diminished (*p* < .05). Nonetheless, numerous lymphocyte levels, including initial Th, initial Tc/s, total Treg, natural Treg, induced Treg, early activated T, and early‐activated types, were akin to controls (*p* > .05). As disease severity amplified, we observed an escalation in middle‐stage activated T lymphocyte levels (*p* < .05) and a decrease in levels of certain lymphocytes such as Breg, T, and aging Tc/s (*p* < .05). Furthermore, the mild patient group exhibited higher B1 and T lymphocyte levels than the healthy controls and other patient categories. As the disease's severity increased, these T lymphocyte levels decreased. Notably, the progression of Tc/s and mid‐stage activated Tc/s lymphocytes displayed a pattern: from mild to severe, then critically ill, and finally the deceased group. On the contrary, B_2_ and late‐activated Tc/s lymphocyte levels progressed in the following order: mild, ordinary, severe, critical, and death groups. The mild and ordinary groups had CD8+ Treg levels exceeding those of the healthy controls. The critically ill patients had fewer early activated Tc/s lymphocytes than the healthy individuals, but Th lymphocyte levels in the mild patient group surpassed those of the deceased group. For T, aging Tc/s, and total aging T lymphocytes, the trend was: healthy controls, ordinary, and then critical groups. Aging Th lymphocyte levels showed an order: mild cases less than ordinary cases, and ordinary cases surpassing severe ones. These observed differences were statistically significant (*p* < .05). However, between patients who died due to COVID‐19 and the critically ill group, the lymphocyte subset expression remained consistent without any significant variance (*p* > .05).

B_1_ cells innately produce antibodies without requiring prior exposure to antigens, offering a first line of defense against airborne pathogens and symbiotic organisms that might breach the intestinal barrier. Patients with mild COVID‐19 often mount rapid immune responses compared to their severely affected counterparts. Meanwhile, B_2_ cells generate high‐affinity antibodies and memory B cells upon antigen exposure, serving as cornerstones of humoral immunity. Our study indicates that as the severity intensifies across groups, an upward trend in COVID‐19 increased, B_2_ cell expression also rises. It is theorized that B_2_ cells, under the guidance of Th cells, jumpstart a humoral immune reaction, producing antibodies to counter the viral invasion. However, in some cases, this response can be overly aggressive, leading to enhanced inflammation and worsening of the patient's symptoms. The recently discovered Breg cells, a subset of B cells, possess immunosuppressive properties. Breg cells play a pivotal role in maintaining immune tolerance, preventing autoimmune disorders, quelling immune inflammation, and releasing anti‐inflammatory agents such as IL‐10 to modulate the body's immune responses.^13^ Our findings reveal that patients with COVID‐19 have a significantly reduced proportion of Breg cells compared with healthy individuals. This deficiency suggests that these patients may be susceptible to enhanced inflammatory reactions. Moreover, we observed elevated levels of activated B lymphocytes in all COVID‐19 patient groups relative to healthy controls. The body's response to COVID‐19 leads to B cell activation, diminishing BCR expression, and their subsequent transformation into plasma cells, which then produce a surge of high‐affinity antibodies.

In our research, both the healthy participants and those with mild COVID‐19 symptoms displayed a notably increased T‐cell expression compared with severely affected patients. This suggests a more potent cellular immune response in these groups. Upon antigen exposure, CD4 + T cells transform into Th cells. Interestingly, Th cell expression levels were considerably higher in patients with mild disease than in deceased patients. A Th1‐mediated response in these mild cases appears to offer a beneficial layer of immunity against infections. This Th‐mediated anti‐inflammatory action could potentially limit rampant inflammation, thus serving a protective role in lung health and homeostasis. By contrast, fatal cases showed a marked disparity in Th cell subset levels, potentially leading to an unregulated immune‐inflammatory response, aggravating the disease, and increasing fatality rates. CD8+ suppressor T cells (Tc/s), known for their regulatory functions, release substances such as perforin‐1, granzyme, and lymphotoxin that cause target cells to undergo apoptosis. We found that Tc/s cell levels were significantly depressed across all COVID‐19 patient categories when compared with healthy individuals. This decrease became more pronounced as the disease's severity escalated, suggesting a waning immune‐regulatory effect. Such patterns might indicate diminishing immune tolerance as the disease intensifies, possibly linked to Tc/s cell depletion. This aligns with prior research by Peng and Mentzer, suggesting that the presence of virus‐specific CD8 + T cells correlates with more favorable outcomes.^7^, ^10^ Furthermore, CD8+ Treg cells, which are inherently immunosuppressive, chiefly modulate activated T cells. They influence the expression of inhibitory receptors such as PD‐1 and CTLA‐4 on T cells and release inhibitory substances. They also engage directly with target cells, tempering excessive immune reactions. Notably, the mild and moderate COVID‐19 patient groups showed pronounced levels of CD8+ Treg cell immunosuppression, surpassing that of healthy controls. This finding suggests enhanced CD8+ Treg cell‐mediated immunosuppression in these groups, likely acting as a countermeasure against unchecked immune inflammation.

T‐cell activation requires two primary signals to elicit a robust immune response. These activation stages are marked distinctly: CD69 is the initial activation marker for T lymphocytes, CD25 indicates intermediate activation, and HLA‐DR is a marker that denotes late‐stage activation.^11^, ^12^, ^14^ Research shows that although T‐cell numbers in patients with COVID‐19 decrease, the remaining T cells exhibit enhanced activation.^15^ Once activated, CD4‐positive T cells can associate with B cells, boosting the latter's viability in the short term. These activated T cells then mobilize to the infection site, playing dual roles: eliminating virus‐infected cells and potentially amplifying the release of T‐cell‐dependent cytokines, reinforcing the immune pathogenesis.^16^ In our study, we found that late‐activated T, Th, and Tc/s lymphocyte expression levels in patients with COVID‐19 were lower than those in healthy subjects. As the disease's severity increased, the presence of intermediate‐activated Tc/s lymphocytes showed a downward trend. This pattern suggests that T cells, once infected with COVID‐19, might be stifled by immunosuppressive cells, hindering their further activation. Such an occurrence could potentially deter T cells from initiating an overly assertive inflammatory reaction. Notably, among patients with severe or critical COVID‐19, levels of early‐activated Tc/s, intermediate‐activated T, and late‐activated Tc/s were markedly higher than in those with milder manifestations of the disease. This heightened T‐cell activation in gravely affected patients might be propelled by a spike in inflammatory cytokines. As a result, there is a noticeable increase in the differentiation of both Th1 and Th2 inflammatory phenotypes, leading to immune imbalances in this critically ill cohort.

Cellular senescence is characterized by a permanent halt in cell proliferation. In response to damage or stress, cells adopt this state to avoid malignancy, while still actively secreting substances that influence their environment. Intense T‐cell proliferation during a viral infection's acute phase can push these cells toward senescence, predisposing them to programmed cell death. However, a balanced interplay between anticytokines and cytokines enables their survival.^17^ Fundamentally, senescent T cells evolve from a subset of effector T cells that resist apoptosis and persist. A pronounced presence of aging‐characteristic CD8 + T cells is observed in older populations, suggesting their role in declining immune functions over time. As immune cells age, they contribute to reduced immune responsiveness. This predisposes older individuals, who are already grappling with age‐related functional reductions, to heightened risks from infections such as COVID‐19. A unique immune regulatory system, characterized by T‐cell dysfunction and inhibitory cytokines such as IL‐10, moderates excessive inflammation and deters expansive immune activation, which could culminate in autoimmune damage.^18^ In our study, both aging Tc/s cells and the overall senescent T‐cell count were lower in patients with COVID‐19 than in their healthy counterparts. Furthermore, as the severity of COVID‐19 increased, the levels of these aging cells decreased. Intriguingly, patients with COVID‐19 with typical symptoms had higher aging Th lymphocyte levels than those with mild or severe infections. This suggests that an increase in senescent T cells in typical COVID‐19 cases might dampen protective immunity and intensify deleterious inflammation.

## 5 CONCLUSION

Our research indicates that a majority of patients with COVID‐19 display elevated levels of B cell and activated B cell expression. By contrast, levels of Breg, Tc/s, activated T, and aging T cells tend to diminish. It is crucial to recognize that T‐cell responses in COVID‐19 cases can be a double‐edged sword—protective in some instances but harmful in others. Moreover, the generation of T cells is not uniform across patients. Their response to SARS‐CoV‐2 peptides can vary, and the cytokine response patterns evolve as the disease progresses. An excessively robust T‐cell response can lead to unintended harm. Critically, gauging how T cells respond to the virus surface antigens might not only provide insights into predicting disease outcomes but also shed light on previously unexplored mechanisms of lymphocyte infection by COVID‐19. Such insights could pave the way for innovative therapeutic approaches.

In our research, we grouped fatalities from all COVID‐19 severity levels under the “death group.” Interestingly, we observed no marked difference in lymphocyte subset expression between this group and those who were critically ill. This finding underscores the relationship between disease outcomes and immune responses. As we grapple with the ongoing pandemic, it is increasingly vital to fortify one's immune system and undergo regular immune assessments. Individuals at higher risk, especially those with pre‐existing conditions, should be periodically updated about their immune status to ensure optimal health. A decline in immunity underscores the need for intervention. The in‐depth analysis of lymphocyte subsets in the peripheral blood of patients with COVID‐19 is of significant clinical relevance. This spans from illustrating the degree of impairment in the immune system to aiding in disease severity evaluation, classification, diagnosis, and prognosis. Prompt monitoring of emerging immune cells can provide clinicians with critical signals about disease trajectory, allowing for more tailored therapeutic strategies. Simultaneously, such observations deepen our comprehension of COVID‐19 pathogenesis on cellular and humoral immunity fronts. This might eventually help identify biological markers predictive of disease severity.

## AUTHOR CONTRIBUTIONS

**Junyuan Huang**: Writing—original draft; writing—review & editing. **Xinqiang Xu**: Data curation; formal analysis; methodology; writing—original draft; writing—review & editing. **Haiqi Zhang**: Data curation; formal analysis; methodology; writing—original draft. **Weiguo Lu**: Conceptualization; funding acquisition; methodology; resources; validation; visualization. **Jiduo Liu**: Conceptualization; data curation; formal analysis; funding acquisition; investigation; methodology; project administration; resources; software; supervision; validation; visualization; writing—original draft; writing—review & editing.

## CONFLICTS OF INTEREST STATEMENT

The authors declare no conflicts of interest.

## ETHICS STATEMENT

All the patients or their families had signed the informed consent. This study was carried out with the approval of the Ethics Committee of the First Affiliated Hospital of Guangzhou University of Chinese Medicine.

## Data Availability

The data sets used and/or analyzed during the current study are available from the corresponding author upon reasonable request.
